# Selection of vaccine-candidate peptides from *Mycobacterium avium* subsp. *paratuberculosis* by *in silico* prediction, *in vitro* T-cell line proliferation, and *in vivo* immunogenicity

**DOI:** 10.3389/fimmu.2024.1297955

**Published:** 2024-01-30

**Authors:** Kari Lybeck, Stig Tollefsen, Heidi Mikkelsen, Siri Kulberg Sjurseth, Claus Lundegaard, Claus Aagaard, Ingrid Olsen, Gregers Jungersen

**Affiliations:** ^1^ Department of Analysis and Diagnostics, Norwegian Veterinary Institute, Ås, Norway; ^2^ National Veterinary Institute, Technical University of Denmark, Kongens Lyngby, Denmark; ^3^ Department of Systems Biology, Centre for Biological Sequence Analysis, Technical University of Denmark, Kongens Lyngby, Denmark; ^4^ Department of Infectious Disease Immunology, Statens Serum Institut, Copenhagen, Denmark

**Keywords:** peptide vaccine, paratuberculosis, *in silico* analysis, MHC binding prediction, CD4+ T-cell lines, IFN-γ, test interference

## Abstract

*Mycobacterium avium* subspecies *paratuberculosis* (MAP) is a global concern in modern livestock production worldwide. The available vaccines against paratuberculosis do not offer optimal protection and interfere with the diagnosis of bovine tuberculosis. The aim of this study was to identify immunogenic MAP-specific peptides that do not interfere with the diagnosis of bovine tuberculosis. Initially, 119 peptides were selected by either (1) identifying unique MAP peptides that were predicted to bind to bovine major histocompatibility complex class II (MHC-predicted peptides) or (2) selecting hydrophobic peptides unique to MAP within proteins previously shown to be immunogenic (hydrophobic peptides). Subsequent testing of peptide-specific CD4+ T-cell lines from MAP-infected, adult goats vaccinated with peptides in cationic liposome adjuvant pointed to 23 peptides as being most immunogenic. These peptides were included in a second vaccine trial where three groups of eight healthy goat kids were vaccinated with 14 MHC-predicted peptides, nine hydrophobic peptides, or no peptides in o/w emulsion adjuvant. The majority of the MHC-predicted (93%) and hydrophobic peptides (67%) induced interferon-gamma (IFN-γ) responses in at least one animal. Similarly, 86% of the MHC-predicted and 89% of the hydrophobic peptides induced antibody responses in at least one goat. The immunization of eight healthy heifers with all 119 peptides formulated in emulsion adjuvant identified more peptides as immunogenic, as peptide specific IFN-γ and antibody responses in at least one heifer was found toward 84% and 24% of the peptides, respectively. No peptide-induced reactivity was found with commercial ELISAs for detecting antibodies against *Mycobacterium bovis* or MAP or when performing tuberculin skin testing for bovine tuberculosis. The vaccinated animals experienced adverse reactions at the injection site; thus, it is recommend that future studies make improvements to the vaccine formulation. In conclusion, immunogenic MAP-specific peptides that appeared promising for use in a vaccine against paratuberculosis without interfering with surveillance and trade tests for bovine tuberculosis were identified by *in silico* analysis and *ex vivo* generation of CD4+ T-cell lines and validated by the immunization of goats and cattle. Future studies should test different peptide combinations in challenge trials to determine their protective effect and identify the most MHC-promiscuous vaccine candidates.

## Introduction

Paratuberculosis is chronic, non-treatable, granulomatous enteritis in ruminants caused by the facultative intracellular bacterium *Mycobacterium avium* subspecies *paratuberculosis* (MAP), which is able to survive and grow inside monocytes and macrophages. Ruminant paratuberculosis vaccines interfere with diagnostics for bovine tuberculosis and paratuberculosis and lack efficacy. There are no exact immunological correlates of protection, but CD4+ T-cells producing interferon-gamma (IFN-γ) to activate intracellular killing by macrophages may indicate an effective immune response against MAP (1–3).

Though animals are often infected at a young age, the clinical signs of MAP are latent and may not become apparent until years after infection (4). The infected animals may have diarrhea and emaciation or subtle signs like reduced milk production, lower reproductive rates, and loss of slaughter weight. Thus, the disease causes both economic and animal welfare concerns (5–7). These concerns are global; for example, in North America and many European countries, over 40% of dairy herds have MAP infections (8, 9). Furthermore, the bacterium has been linked to the development of Crohn’s disease in humans, leading to increased interest in effective MAP control strategies (10–12).

Paratuberculosis control strategies based only on testing and culling have generally been unsuccessful (13, 14). This is partly because the bacteria can survive for over a year in the environment (15) and partly due to the multi-year incubation period for paratuberculosis as the sensitivity of diagnostic tests is low during the subclinical stages. Vaccination against MAP may help control paratuberculosis. Commercially available MAP vaccines generally consist of live attenuated or killed whole bacteria with a mineral oil adjuvant. These vaccines reduce the clinical signs and shedding of MAP, but they do not prevent all animals from infection and subsequent transmission of the bacteria (2, 16, 17). Additionally, these vaccines may cause false-positive reactions on immune-based tests for *Mycobacterium bovis* (16, 18), just as there is evidence that co-infections with *M. bovis* and paratuberculosis may reduce the sensitivity of the tuberculin skin test and the IFN-γ release assay used for detecting bovine tuberculosis (19, 20). The development of a MAP vaccine providing improved protection while not interfering with the diagnosis of *M. bovis* and preferably also paratuberculosis is therefore desirable.

In addition to inducing cross-reactivity in tests for *M. bovis*, whole-cell vaccines may also have other disadvantages. When using live attenuated vaccines, live organisms are shed into the environment and could potentially revert to a virulent form. In vaccines with killed organisms, antigens essential for protection may have been removed or altered in a way that they are no longer immunogenic (17, 21). Furthermore, whole-cell vaccines contain a range of proteins that are irrelevant for protection (22), and the cell wall of MAP may even have immune regulators that could interfere with the development of a protective immune response (23).

Peptide vaccines designed to induce T-cell responses activated by bacterially expressed proteins are attractive because they only include antigens regarded as important for protection and because peptides can be produced synthetically at a low cost and a large scale (24). Additionally, by including only MAP-specific peptides, false-positive tests for *M. bovis* due to vaccination may be avoided. The genome of the MAP cattle-type strain is available from the K-10 isolate (25, 26), and the complete sequence of another bovine strain, JII-1961, has more recently also become available (27). Genomic tools can be used to compare the MAP genome to other mycobacterial genomes, especially *M. bovis*, facilitating the identification of MAP-specific peptides. If the peptides included in a vaccine are different from the antigens used in diagnostic tests, the MAP-infected and MAP-vaccinated animals can be differentiated (DIVA strategy). Subunit vaccines generally have not provided the desired level of protection against MAP (17, 28, 29), though there is some evidence of partial protection after the vaccination of calves with four recombinant MAP antigens (30). Although the immunogenicity of peptides might be low, the inclusion of suitable adjuvants, stabilizers, or carriers in the vaccine may help (31). A recent study found that the vaccination of cattle with a soluble, recombinant fusion protein based on four MAP-antigens in o/w emulsion adjuvant induced strong antigen-specific IFN-γ, IL-17, and antibody responses, without interfering with skin testing for bovine tuberculosis (32).

The pathogenesis of intracellular organisms like MAP is complex, and a range of different virulence factors are involved (33). To increase the chances of developing an effective peptide vaccine against MAP, it is likely that peptides from multiple proteins must be included in order to induce immune responses that are appropriate at different time points during infection where mycobacterial protein expression may vary. The use of multiple peptides will also improve the chances of successful immunization in a population with highly diverse major histocompatibility complex (MHC) molecules (34) as well as reduce potential negative consequences on vaccine efficacy due to epitope mutations (35).

To develop subunit vaccines, the immunogenicity of candidate antigens must be evaluated to ensure the binding of a peptide to the MHC class II molecule to initiate adaptive CD4+ T helper 1 (Th1) immune responses. By using algorithms, potential T-cell epitopes can be identified *in silico*, leading to reduced laboratory time, higher cost efficiency, and less use of animal experiments. This method has been used with success to find vaccine antigens for *Mycobacterium tuberculosis* (36) and thus may help identify novel MAP vaccine candidates (37, 38). Importantly, effective methods for predicting peptide binding to bovine (BoLA) DR alleles have been developed (39, 40). Following immunization or infection, identification of CD4+ T-cells of low abundance responding to a specific, immunogenic antigen is difficult and relevant antigens may go undetected when examining antigen–T-cell interactions *in vitro*. One way to overcome this is to characterize antigen-specific T-cell responses through establishment of T-cell lines and T-cell clones, as this will increase the number of antigen-specific cells. In humans, screening of a genomic *Mycobacterium tuberculosis* (*M. tuberculosis*) library by the use of CD4+ T-cell lines has been important in the discovery of potential vaccine antigens that were recognized by multiple T-cell lines from several donors (41).

The aim of this study was to identify immunogenic peptides to include in a subunit vaccine providing improved protection against MAP without interference with the diagnosis of bovine tuberculosis. We describe the identification of 119 MAP-specific peptides by the use of *in silico* analysis with *in vivo* immunogenicity assessment by IFN-γ and antibody responses following the vaccination of goats and cattle and the generation of peptide-specific CD4+ T-cell lines.

## Materials and methods

### Animals in the study

This study consisted of three different immunization trials with three different sets of animals.

MAP-infected goats: Three female Norwegian dairy goats naturally infected with MAP were transported to the Laboratory Animal Unit at the Norwegian Veterinary Institute (Oslo, Norway) for immunization. Shedding of MAP was confirmed in all three goats by fecal culture upon arrival from the field. The goats were 4 to 5 years old when immunized, and the trial lasted for 8 weeks.

Healthy goat kids: A total of 24 healthy, castrated, male Norwegian dairy goat kids from the Animal Production Experimental Centre, located at the Norwegian University of Life Sciences (NMBU, Ås, Norway), remained in the immunization experiment from 3 to 5 months of age. The kids were divided into three immunization groups but were housed together during the trial. Seven goats were kept for an additional 3 months to generate T-cell lines.

Healthy cattle: Nine Norwegian red heifers, 6–12 months of age, were from a farm in the southeastern part of Norway (Oslo). The heifers were kept on the farm of origin during the immunization trial that lasted for 8 weeks.

All animals were kept indoors during the trials. None of the animals had been vaccinated against paratuberculosis. Neither goats nor heifers were MHC-genotyped. The healthy goat kids and heifers were from an area of Norway with no history of MAP infection.

### Selection of unique MAP peptides

The genome of two MAP strains (K-10 and a Danish Ejlskov2007 strain) were used as “positive” strains and compared to five “negative” mycobacterium species genomes: (*M. tuberculosis* (H37Rv and CDC1551), *M. bovis* (BCG Pasteur 1173P2 and AF2122/97), and *M. avium* (strain 104). All assigned reading frames from the compared mycobacterial genomes were virtually translated into protein sequences and converted into every possible sub-peptide of 20 amino acids in length. Peptides from the Ejlskov2007 strain that had an identical match in the K-10 strain were selected as potential positive hits. All selected peptides were compared to all 20mers from each of the negative strains and discarded if a common motif of eight consecutive amino acids was found. This filtering resulted in approximately 80,000 20mers 100% conserved between the two positive strains, with no 8mer overlap in any negative genome. All residual 20mers were now *in silico*-checked for predicted binding to bovine class-II MHCs BoLA-DRB3*0101, -DRB3*1101, -DRB3*1201, -DRB3*3001, and -DRB3*4501 using NetMHCIIpan-2.1. A total of 1,121 peptides were predicted to bind to all five MHCs, and 113 of these had unique binding cores. The 59 peptides with the highest predicted binding affinity averaged over all five MHC alleles were selected for experimental validation. These peptides were termed “MHC-predicted peptides”. The MHC-predicted peptides were also tested for predicted binding to goat MHC class II beta chains and found to bind promiscuously. In addition, 60 peptides were selected from a limited number of proteins, previously shown to be immunogenic. As hydrophobicity has been shown to correlate with the immunogenicity of peptides (42), 20mer peptides from the limited set of proteins were scored by hydrophobicity and selected for MAP specificity (presence in positive genomes and absence in negative genomes). These peptides were termed “hydrophobic peptides”. In total, 119 peptides were identified for further testing. The sequences and proteomic origin of the identified MAP-specific peptides are shown in **Tables 1**, **2**. The peptides were checked for comparison with MAP peptides registered in the Immune Epitope Database (IEDB) by other studies. Only nine bovine MAP peptides and no caprine MAP peptides were registered in the IEDB, and none of them had a sequence identical to the peptides identified in this paper. Finally, BlastP analysis for molecular mimicry, in general, showed that a maximum of seven consecutive amino acids were similar between the identified peptides and the bovine or caprine genome. Given that up to 99.7% of bacterial heptapeptides are shared between microbes and humans (43), this overlap was accepted. The exception was one peptide (no. 52) that had eight consecutive amino acids in common with a few peptides in the bovine or caprine genome. This peptide should thus not be included in a future vaccine.

**Table 1:** *In silico*-selected MAP-specific hydrophobic peptides.

Hydrophobic peptides									
Peptide name	Peptide number (peptide pool)	Sequence	Length	Mw (mg/mmol)	Peptide name	Peptide number (peptide pool)	Sequence	Length	Mw (mg/mmol)
MAP3779-1	1 (1)	LLVTGLAGCFVFSLI	15	1,552.95	MAP3272-3	31 (4)	VGAWSVIIRGIARSL	15	1,597.94
MAP3779-2	2 (1)	IPAPRPIALGLFNAI	15	1,562.93	MAP3272-4	32 (4)	KLISAAINNRVLFEV	15	1,687.03
MAP3779-3	3 (1)	RAVAMGAVLLVTGLA	15	1,441.81	MAP3272-5	33 (4)	LLRGLTLGRLVTHAD	15	1,634.76
MAP3779-4	4 (1)	LYVRVGEDLHPVLNL	15	1,737.05	MAP2888-1	34 (4)	YDLLRRNQILFTFLH	15	1,949.3
MAP3779-5	5 (1)	QALLADRDGTTWLLW	15	1,759.01	MAP2888-2	35 (4)	MVTVLDVNINKLRLL	15	1,741.19
MAP3779-6	6 (1)	WSVLSLFASGPTLSR	15	1,620.88	MAP2888-3	36 (4)	IGAVLLPGAKAPNLI	15	1,446.81
MAP3785-1	7 (1)	FAIAVAIEHLTALLP	15	1,578.93	MAP2888-4	37 (4)	MPYVLALADRGWRAA	15	1,690.01
MAP3785-2	8 (1)	LINPAVAEWIKVVCF	15	1,702.1	MAP_0165	38 (4)	FLGTVAAGVLVLAIA	15	1,414.77
MAP3785-3	9 (1)	LPLPLIAGYLDRYGI	15	1,674.03	MAP_1507	39 (4)	LAAAAGNLQAIGWTL	15	1,469.72
MAP3785-4	10 (1)	IKVVCFPQRWLDLRY	15	1,936.36	MAP_3783	40 (4)	FVEVAARVNTLLDIA	15	1,630.92
MAP3785-5	11 (2)	VVALRSAQLITFTAM	15	1,620.99	MAP1588c-1	41 (5)	LKAAAIISGVAQAIV	15	1,424.76
MAP3785-6	12 (2)	LPALTALDLLSTGRL	15	1,553.88	MAP1588c-2	42 (5)	KANFELWCFAVSAIN	15	1,713
MAP3785-7	13 (2)	LVPVLTVGLAQRAPA	15	1,504.85	MAP1588c-3	43 (5)	ALGAASVMAMNNVFY	15	1,558.85
MAP3785-8	14 (2)	WIGLTVSAPDNLAAL	15	1,504.8	MAP2768c-1	44 (5)	LRLMVALASWCALRF	15	1,750.21
MAP3785-9	15 (2)	WFIAETTGAGSFPWV	15	1,668.88	**MAP2768c-2**	**45 (5)**	**LPILTEAMPERLRLM**	**15**	**1,783.24**
MAP3785-10	16 (2)	LIPQRGNHLPALTAL	15	1,613.94	MAP2768c-3	46 (5)	VAIPPHLVSAIEAHL	15	1,566.88
MAP3651c-1	17 (2)	WIEQMKRIGIYGLAV	15	1,777.18	MAP2768c-4	47 (5)	ILDDHLLPAFGSRQL	15	1,694.97
MAP3651c-2	18 (2)	GISILLVEHGPGLTV	15	1,504.8	**MAP2557c-1**	**48 (5)**	**IGMPNSEELIITTLL**	**15**	**1,643.97**
MAP_2487c	19 (2)	VYRILGLREGEAHVI	15	1,725.05	MAP2557c-2	49 (5)	LIITTLLSPSSMSHA	15	1,570.88
MAP_3701c	20 (2)	LALWTRPAWDTDRWL	15	1,900.19	**MAP1693c-1**	**50 (5)**	**GIRPGDTLVFAIKIL**	**15**	**1,612.99**
MAP0693-1	21 (3)	PAEYGSIIAPTLVLW	15	1,629.93	MAP1693c-2	51 (6)	FPLGGVVPGFQKAIA	15	1,500.82
MAP0693-2	22 (3)	PVVMQRIIALSMAAA	15	1,570.99	MAP1511-1	52 (6)	WVAAGGLFGALLIGG	15	1,401.68
**MAP0693-3**	**23 (3)**	**PLEVRHYVEHLMTVL**	**15**	**1,836.2**	MAP1511-2	53 (6)	IPKPVPHRDMVPIWV	15	1,784.22
MAP0693-4	24 (3)	AAHADHFWTWSIDML	15	1,801.03	MAP1050c-1	54 (6)	ILLIAAGSIVAVAVI	15	1,422.83
MAP0693-5	25 (3)	AKTFNRLHLDFLLGR	15	1,801.14	MAP1050c-2	55 (6)	IVAVAVIAAVVVTVL	15	1,436.84
MAP0693-6	26 (3)	YLRAGDPGKPVLVLL	15	1,610.98	MAP_1589c	56 (6)	QFPAYELTALIAGDL	15	1,621.87
**MAP0693-7**	**27 (3)**	**PTWETVQARIKWLMA**	**15**	**1,830.2**	**MAP_1653**	**57 (6)**	**LVLNIIPSIDTPVCA**	**15**	**1,567.91**
**MAP0693-8**	**28 (3)**	**LMTVLRTIGVERASI**	**15**	**1,659.04**	MAP1662c-1	58 (6)	VTDVVLAAPIGAFGF	15	1,476.75
**MAP3272-1**	**29 (3)**	**IFPVNYVVQRRTVLF**	**15**	**1,851.24**	MAP1662c-2	59 (6)	LPPGVDQHIAAVALF	15	1,547.83
**MAP3272-2**	**30 (3)**	**LPVRECWDLLRGLTL**	**15**	**1,784.16**	MAP1662c-3	60 (6)	VAMAAALLLPPGTPG	15	1,378.71

The peptide name indicates the MAP gene of origin (open reading frame from https://www.ncbi.nlm.nih.gov/nuccore/AE016958), followed by running number. Peptides in bold blue indicate peptides chosen for further immunization based on the proliferation of CD4+T-cell lines from MAP-infected, peptide-vaccinated goats. Each peptide was assigned to a peptide pool (1–6) for practical reasons during testing as indicated in brackets.

**Table 2:** *In silico*-selected MAP-specific MHC-predicted peptides.

MHC-predicted peptides									
Peptide name	Peptide number (peptide pool)	Sequence	Length	Mw (mg/mmol)	Peptide name	Peptide number (peptide pool)	Sequence	Length	Mw (mg/mmol)
MAP3742-b	61 (7)	LLRAMVSDPTLAAAA	15	1,499.81	**MAP2959c**	**91 (10)**	**VDGIVHALRSILAEA**	**15**	**1,563.83**
MAP3742-a	62 (7)	NVAIVTAFSRAIARY	15	1,651.94	MAP2943c	92 (10)	LGRRLHRALGPLVHVN	16	1,808.18
MAP3437c	63 (7)	ELIGFARALSVQTAT	15	1,576.83	**MAP2740**	**93 (10)**	**VEPLLHSIPPLAVYLV**	**16**	**1,760.17**
MAP2544c	64 (7)	PVTGIARSLAILAAS	15	1,439.73	MAP2324c	94 (10)	KYVLRRALPVGLSVV	15	1,670.09
MAP4333	65 (7)	AIIKALRTAAVNDTK	15	1,584.89	MAP2189	95 (10)	RRPTFRYARPLAGLA	15	1,745.08
MAP4303c	66 (7)	SGYVKFDRAAALRRL	15	1,723.03	MAP2188c	96 (10)	PKELRYILSSVRPRVFIT	18	2,174.64
MAP4266	67 (7)	NETYVWSRTARIRQL	15	1,893.15	MAP2100	97 (10)	VARREIRLLLANRLYFAF	18	2,221.7
MAP4005	68 (7)	PRRLRSTPALRRLVA	15	1,762.15	MAP2070	98 (10)	LARTLRRALPLMARLT	16	1,852.34
MAP3836c	69 (7)	QELHAIIKALRTAAVNDT	18	1,964.27	MAP1857	99 (10)	WRRYLRRSVLPLLLA	15	1,912.38
MAP3818	70 (7)	SDLINGIRSMPVRFT	15	1,706.01	MAP1720	100 (10)	RSSVIRLAPTVHGPG	15	1,546.81
MAP3814c-b	71 (8)	EPLRRLVKIRSSIVKRRT	18	2,207.72	MAP1677	101 (11)	MPTLRLGRAARVAVL	15	1,624.04
**MAP3814c-a**	**72 (8)**	**GEPLRRLVKIRSSIV**	**15**	**1,723.11**	MAP1620	102 (11)	QRVLVRGARARLVAV	15	1,664.05
MAP3776c	73 (8)	YGQLVQLAKALHVAV	15	1,609.95	**MAP1348c**	**103 (11)**	**LLLRFTAAPPASVPS**	**15**	**1,539.85**
**MAP3773c-b**	**74 (8)**	**TSVYRILRALAADRIAET**	**18**	**2,019.35**	MAP1344	104 (11)	VPFTMVAAAPIRAMV	15	1,574
**MAP3773c-a**	**75 (8)**	**LTSVYRILRALAADR**	**15**	**1,718.05**	MAP0970	105 (11)	LQTVFHHGRSLLIAT	15	1,693
MAP3772c	76 (8)	SVTFRAARPLHPQRL	15	1,749.07	MAP0957c	106 (11)	PGFITMNLPRLLDAL	15	1,671.05
MAP3764c	77 (8)	VRRIRAYASARTAHY	15	1,791.06	MAP0954	107 (11)	GPLIRLASAKGFRVIV	16	1,697.12
**MAP3761c**	**78 (8)**	**GIALLLISRSRPVLNLLA**	**18**	**1,919.4**	MAP0917	108 (11)	RAPLRATPSLSLRWR	15	1,780.13
**MAP3758c**	**79 (8)**	**HSIEMLRVHPARSVS**	**15**	**1,719.01**	**MAP0860c**	**109 (11)**	**LAVLAMSPAARFAEK**	**15**	**1,574.92**
MAP3754	80 (8)	TPEQIVRKLTAARLRAAG	18	1,951.32	**MAP0763**	**110 (11)**	**LENILHAVPNALGNF**	**15**	**1,621.87**
MAP2192-a	81 (9)	GSFIINNLSNPVHFF	15	1,705.95	MAP0652	111 (12)	IAVFLLDRAVPALQR	15	1,682.06
MAP1753	82 (9)	VYLVVVSSTPIALVA	15	1,530.88	MAP0147c	112 (12)	RIRRALTTLARRVVI	15	1,794.24
MAP2192-b	83 (9)	VENILHAAPTAFSNG	15	1,540.71	MAP0087	113 (12)	ATRVSVLRSAIAPLISP	17	1,751.12
MAP2184c	84 (9)	KGGVIQLTRAVAIEA	15	1,525.83	MAP0014	114 (12)	QLQRVHWRLSAHALI	15	1,828.17
MAP3750	85 (9)	TLSVITTLPSMSVNV	15	1,561.87	**MAP1723-a**	**115 (12)**	**PGARVIKAFNTLHARYII**	**18**	**2,040.46**
MAP3749	86 (9)	KKVIRDYTRSLALTL	15	1,777.16	MAP0963c	116 (12)	LERLVRINSGLALLY	15	1,730.1
MAP3747c	87 (9)	SIESILRNLHPLARRG	16	1,832.16	**MAP0092**	**117 (12)**	**GLAVIRQAASLVAAV**	**15**	**1,438.75**
**MAP3744-a**	**88 (9)**	**SLARIRYVHARASLH**	**15**	**1,750.05**	MAP3744-b	118 (12)	LARIRYVHARASLHVVYP	18	2,121.54
MAP3731c	89 (9)	IADRIVLLRNGRIAA	15	1,651	MAP1632c	119 (12)	RSIFLINIPAAAIIA	15	1,582.96
**MAP3481**	**90 (9)**	**KRSMIRKVSVALAVL**	**15**	**1,671.14**					

The peptide name indicates the MAP gene of origin (open reading frame from https://www.ncbi.nlm.nih.gov/nuccore/AE016958), followed by running letter. Peptides in bold blue indicate peptides chosen for further immunization based on the proliferation of CD4+T-cell lines from MAP-infected, peptide-vaccinated goats. Each peptide was assigned to a peptide pool (7–12) for practical reasons during testing as indicated in brackets.

### Production of peptides

Synthetic, lyophilized peptides (15–18 amino acids) produced by EZBiolab (Carmel, USA) or JPT Peptide Technologies (Berlin, Germany) with >90% purity and no C- or N-terminal modifications were dissolved dropwise in dimethylformamide (DMF) or dimethyl sulfoxide (DMSO) before transferring dropwise into phosphate-buffered saline (PBS) assuring DMF or DMSO concentrations <0.1% during testing.

### Animal immunogenicity studies

Firstly, three naturally MAP-infected adult goats were immunized twice at a 4-week interval with all 119 peptides (50 μg of each, final concentration) formulated with CAF 04 liposome adjuvant containing dimethyl dioctadecyl ammonium bromide-monomycolyl glycerol (44, 45).

The 23 peptides inducing the strongest T-cell line responses in MAP-infected goats were included in a second vaccine trial with 24 healthy goat kids. The kids were randomly allocated into three groups of eight animals and immunized twice at a 5-week interval with 14 MHC-predicted peptides (grp. 1), nine hydrophobic peptides (grp. 2), or PBS only (grp. 3) in Montanide ISA61 VG (Seppic, Puteaux, France). Due to low immune responses in the initial trial with MAP-infected goats, immunization with 20 µg of each peptide was tested in this trial (46). One goat in the hydrophobic peptide group died during the experiment due to an accident unrelated to vaccination.

Finally, the immunogenicity of the peptides was examined in cattle by immunization of eight healthy heifers with all 119 peptides (20 µg/peptide) in Montanide ISA61 VG. The heifers were immunized only once. One additional animal was injected with adjuvant only. Details on the vaccine trials are shown in **Supplementary Table S1**.

### Generation of T-cell lines

A protocol was established for the generation of T-cell lines from goats and cattle to evaluate the immunogenicity of the 119 MAP-specific peptides. In this work, a T-cell line was defined as T-cells from a particular animal after cultivation with one or more antigens over a 16- to 17-day period. The protocol was first optimized for the generation of T-cell lines from MAP-infected goats cultured with purified protein derivative from MAP (PPDj) (used in Lybeck et al. (47)). Next, peptide-reactive CD4+ T-cell lines were generated from the blood of MAP-infected goats after peptide vaccination. Finally, peptide-reactive CD4+ T-cell lines were generated from healthy goats and heifers following peptide immunization.

Preparation of non-proliferating feeder cells: Peripheral blood mononuclear cells (PBMCs) were isolated using gradient centrifugation with Lymphoprep (Axis-Shield, Oslo, Norway), incubated for 1.5 h in RPMI medium 1640 (RPMI) (Life Technologies, Carlsbad, USA) with 10% heat-inactivated fetal calf serum and 10 µg/mL Mitomycin C (Sigma Aldrich, St. Louis, USA), and washed three times in PBS de Boer (pH 7.4). As an alternative to Mitomycin C treatment, irradiation of PBMCs (1.2 × 10^6^ in 2 mL RPMI) at a dose of 11.7 Gray using a RS2000 biological irradiator (Rad Source Technologies Inc., Buford, USA) was introduced for the later experiments.

Cultivation of T-cells: The protocol for the cultivation of T-cells was derived from studies in human (10, 48). Initially, mixed T-cell lines without prior selection of CD4 T-cells were generated by cultivation of PBMC. However, due to the overgrowth of CD8+ and γδ TCR+ T-cells with this protocol, it became necessary to affinity-purify CD4+ T-cells from PBMC (20 × 10^6^) using a monoclonal antibody against CD4 (**Supplementary Table S2**) and Dynabeads Pan Mouse IgG (Invitrogen) according to the manufacturer’s instructions. The CD4-selected T-cells were dissolved in 1 mL RPMI with 1.2 × 10^6^ autologous feeder cells, 10% heat-inactivated goat serum, 10 IU gentamycin, 50 µM 2-mercaptoethanol, and non-essential amino acids (1:100) (all Life Technologies) and seeded into U-bottom 96-well plates. The cells were incubated with 5 µg/mL PPDj or pools of nine to 10 peptides (5 µM/peptide) in addition to 1 ng/mL recombinant human IL-15 (eBioscience, San Diego, USA) and 50 U/mL of recombinant ovine IL-2 for the goats experiments (49) or 1.75 ng/mL recombinant human IL-2 for cattle experiments (eBioscience). The T-cells were restimulated after 8 days with allogeneic non-proliferating feeder cells, IL-2, IL-15, and phytohaemagglutinin (PHA, 1 µg/mL, Thermo Fisher Scientific, Lenexa, KS, USA) and then grown for another 8 to 9 days. During cultivation, the T-cells were split depending on the growth speed and fresh medium with IL-2 and IL-15 was added.

Proliferative T-cell assay: After 16 to 17 days of cultivation, T-cell lines were tested for recall responses toward PPDj or the peptides used for stimulation. Autologous PBMCs were added to F-bottom 96 well plates (1.2 × 10^5^ cells/well) and incubated for 1.5 h before washing away non-adherent cells and adding antigens: PPDj (5 µg/mL), pools of nine to 10 peptides (5 µM/peptide), *E. coli* as control antigen (5 µg/mL) (10), or media only. The plates were incubated overnight before adding the cultivated T-cells (50,000–80,000 cells/well). After incubation for 72 h, CellTiter-Glo reagents (Promega, Madison, WI, USA) were added according to the manufacturer’s instructions. Proliferation was calculated as % change *versus* control = [(mean luminescence antigen-stimulated wells/mean luminescence unstimulated wells) -1] × 100. Peptides were classified as inducers of T-cell line responses when the % change *versus* control was ≥20, and values at ≥20 to <50 were considered medium responses. Values ≥50 were classified as strong responses. Responses ≥100 were further classified as very strong. The T-cell lines with the strongest responses against pooled peptides were expanded with allogeneic feeder cells, PHA plus cytokines as above, and grown for approximately 10 days before testing for recall responses toward individual peptides (5µM).

Phenotyping of surface markers: T-cell lines were stained in 96-well plates (1 × 10^5^ cells/well) with unconjugated monoclonal antibodies (mAbs) against CD4, CD8, and γδ TCR, incubated with appropriate secondary antibodies (**Supplementary Table S2**), and analyzed on a FACSCalibur flow cytometer (Becton Dickinson, San Jose, CA, USA) with the CellQuest Pro software, version 6.0.2, and later with a Novocyte flow cytometer (ACEA Biosciences, San Diego, USA), with NovoExpress software, version 1.2.4 (ACEA biosciences, San Diego, USA). Positive fluorescence gates were set with reference to negative controls where primary antibodies were omitted.

Intracellular staining for cytokines: Adherent PBMCs were stimulated for 18 h with PPDj (5 µg/mL) or the positive controls 0.5 μg/mL *Staphylococcus aureus* enterotoxin D (SED), (Toxin Technology, Sarasota, USA) or 10 μg/mL Concanavalin A (ConA), (Sigma Aldrich, St. Louis, USA). The protocol for intracellular staining was as described earlier (50) with the addition of Brefeldin A after 6 h of stimulation. Briefly, cells were stained with surface markers as for phenotyping, fixed, permeabilized, and stained with primary mAbs against IFN-γ and subsequently with PE-conjugated secondary antibodies before analyses as described above (**Supplementary Table S2**).

### IFN-γ assay

Heparin-stabilized blood was stimulated with peptides either individually or as pools of nine to 10 peptides (2 µM/peptide), PPDj (10 µg/mL), SED (0.5 µg/mL), or no antigen in F-bottom 48- or 96-well plates. After 24 h, plasma was harvested, stored at -20°C, and analyzed by a capture enzyme-linked immunosorbent assay (ELISA) for bovine IFN-γ according to the manufacturer’s instructions (Life Technologies). OD values were read at 450 nm, and ΔOD values were calculated by subtracting the IFN-γ response in wells with no antigen from the response in the antigen-stimulated wells. A twofold dilution series of recombinant bovine IFN-γ (AbD Serotec, Oxford, England) was included on plates for the calculation of IFN-γ concentrations (ng/mL). Based on experience with IFN-γ testing of goats for MAP in a Norwegian sanitation program (47), the cutoff for a peptide to be classified as resulting in an IFN-γ response was peptide-stimulated – unstimulated response (ΔOD) ≥0.2, corresponding to 0.44 ng/mL (weak response). Values ≥3.9 ng/mL were classified as strong responses. For cattle, IFN-γ values ≥1.8 ng/mL were classified as positive responses. Values between 7 and 14.7 ng/mL were classified as medium responses, while values ≥14.7 were classified as strong responses.

### In-house ELISA for the detection of IgG against MAP-specific peptides

Microtiter plates (Maxisorp for goats and Polysorp for Cattle, Nunc A/S, Roskilde, Denmark) were coated overnight at 4°C with 2 µM of individual peptides in 50 µL PBS, blocked with 200 µL PBS with 1% bovine serum albumin (BSA), washed × 3 (PBS with 0.1% Tween 20), and incubated for 1.5 h with 50 μL serum (1:400 diluted in PBS with 0.2% BSA and 0.2% Tween 20, dilution buffer). After washing, plates were incubated with 50 µL peroxidase-conjugated monoclonal anti-goat/sheep IgG (clone GT-34, Sigma-Aldrich) cross-reacting with bovine IgG, diluted 1:5,000 (goat assay) or 1:10,000 (cattle assay) in dilution buffer before adding 100 µL 3,3′,5,5′-tetramethylbenzidine (Thermo Fisher Scientific, USA). The reaction was stopped with 50 uL of 2 M H_2_SO_4_ (concentrate from Sigma Aldrich), and absorbance was read immediately at 450 nm. Pooled samples from all healthy goats at day 0 were used as a negative control, while pools of samples at week 6 functioned as positive control. Similarly, negative and positive controls were made from all vaccinated cattle by pooling serum from day 0 and week 2, respectively. Plate-to-plate calibrated OD (ODc) was calculated as follows: [(ODSample − ODNegC) × (ODPnorm − ODNnorm)/(ODPosC − ODNegC)] + ODNnorm. ODSample is the sample OD, ODPosC is the positive control, ODNegC is the negative control, ODPnorm is the mean of positive control of all plates, and ODNnorm is the mean of all negative controls. The cutoff for a peptide to be classified as inducing an antibody response was calculated based on the arithmetic mean ODc of pre-immune sera plus three standard deviations, giving an estimated cutoff at ODc 0.2 for goat samples and 0.36 for cattle samples. In addition, ODc values ≥1.0 were classified as strong antibody responses.

### Tuberculin skin testing of vaccinated cattle

Skin testing of cattle was performed 8 weeks after peptide vaccination with the single intradermal comparative cervical tuberculin (SICCT) test. The avian**-**purified protein derivative (PPDa) and bovine-purified protein derivative (PPDb) used for skin testing were from Prionics, Lelystad BV (Lelystad, Holland). The test was performed by injecting 0.1 mL PPDa (2,500 IU) and 0.1 mL PPDb (3,000 IU) intradermally in the neck. Skin thickness was measured at injection and after 72 h to evaluate if an increase had occurred. The procedure was performed and the results interpreted according to standard protocol (European Communities Commission regulation 141 number 1226/2002) (51).

### ELISA for the detection of antibodies to MAP and *M. bovis* in vaccinated cattle

Serum from cattle at day 0 and 2, 4, and 6 weeks after vaccination was analyzed with the ID Screen paratuberculosis Indirect ELISA (IDvet, Grables, France) and the IDEXX *M. bovis* antibody test (Hoofddorp, The Netherlands; analyzed at Animal and Plant Health Agency, Starcross, UK). Both tests were performed according to the manufacturer’s instructions, respectively.

### Statistical analysis

Data were analyzed using the statistical software GraphPad Prism 9.0.1. Wilcoxon matched pair signed-rank test (two-tailed, *P* ≤ 0.05) was used to calculate the significance for T-cell proliferation and IFN-γ production by flow cytometry. Differences between groups for IFN-γ and antibody responses toward peptide pools or individual peptides were compared using the Mann–Whitney *U*-test (two-tailed, *P* ≤ 0.05), while Wilcoxon matched pair signed-rank test (two-tailed, *P* ≤ 0.05) was used to compare IFN-γ and antibody responses before and after vaccination.

## Results

**Figure 1:**
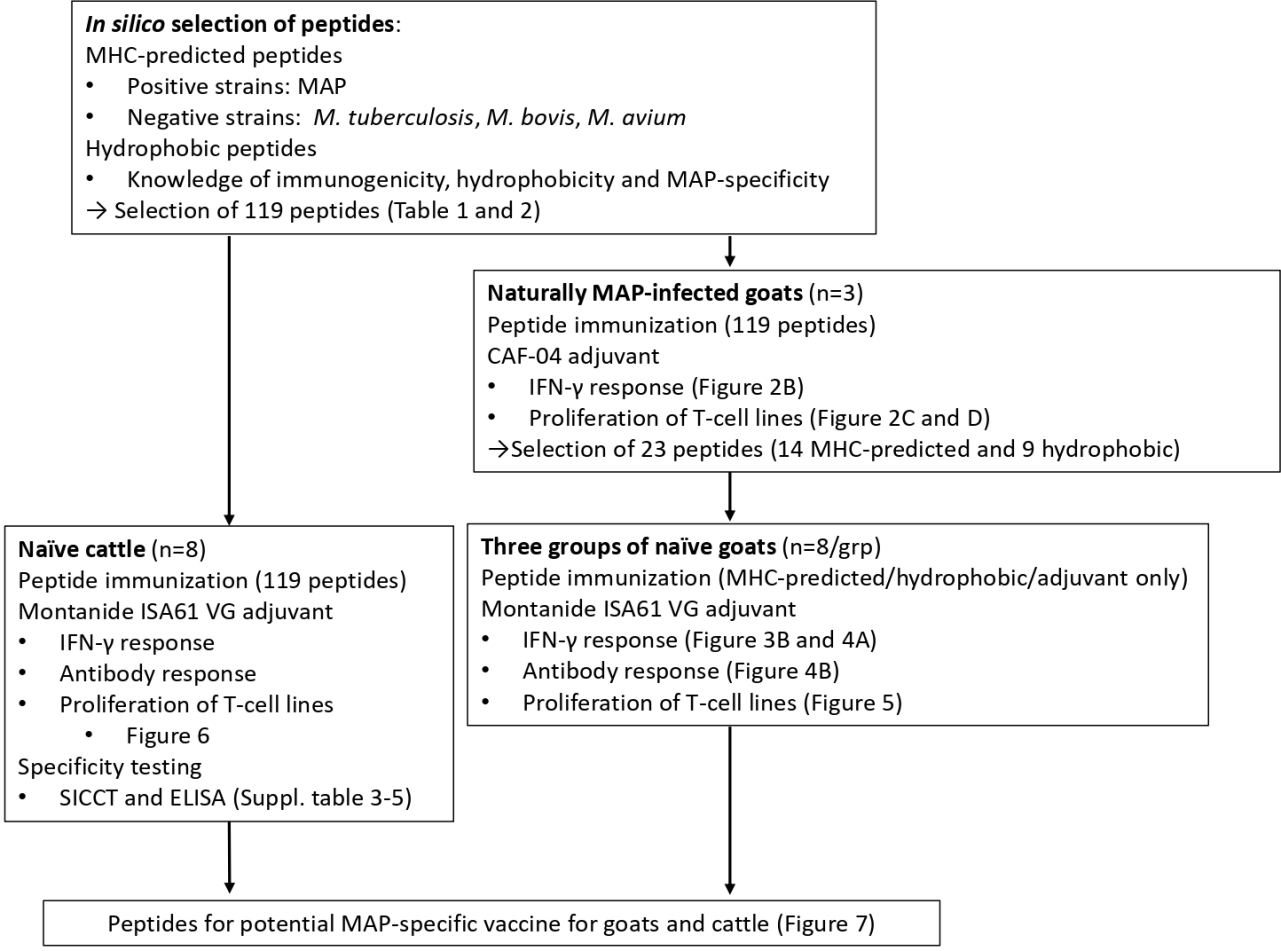
Overview of the study. One panel of MAP-specific peptides was identified by comparing two MAP genomes (positive strains) with five other mycobacterial genomes (*M. tuberculosis*, *M. bovis*, and *M. avium*; negative strains) and the use of MHC class II prediction binding (MHC-predicted peptides). The other peptide panel was identified by taking into account previous knowledge of immunogenicity, hydrophobicity, and MAP specificity (hydrophobic peptides). Following vaccination of MAP-infected goats with peptides formulated with CAF04, 14 MHC-predicted and nine hydrophobic peptides were identified as most immunogenic. The immunogenicity of peptides was further assessed in healthy goats and cattle after vaccination with peptides in Montanide ISA 61 VG by measuring IFN-γ production, antibody responses, and proliferation of T-cell lines. Taken together, the study proposes peptide pools that could be included in future vaccines for goats and cattle.

An overview of the study and screening strategy is shown in **Figure 1**.

### Injection site reactions in goats and cattle

The vaccination of MAP-infected goats with CAF04 liposome adjuvant did not result in injection site reactions. Vaccines were injected subcutaneously in the axilla of goat kids. Diffuse swelling and granuloma-like structures up to 3 to 4 cm in diameter were seen at the injection site less than 1 week after vaccination. Reactions were seen in goats vaccinated with either MHC-predicted or hydrophobic peptides, while no reactions were seen in goats injected with adjuvant only. The reactions had diminished after 4 weeks but increased again upon revaccination. The reactions disappeared gradually thereafter. The cattle received 59 or 60 peptides on the left and right side of the neck, respectively. The animals developed injection site reactions similar to goats, but of a larger size (up to 11 cm). The reactions persisted throughout the trial period. No sign of infection was seen at the injection site in either goats or cattle.

### IFN-γ and T-cell line responses of MAP-infected goats immunized with MAP-specific peptides

**Figure 2:**
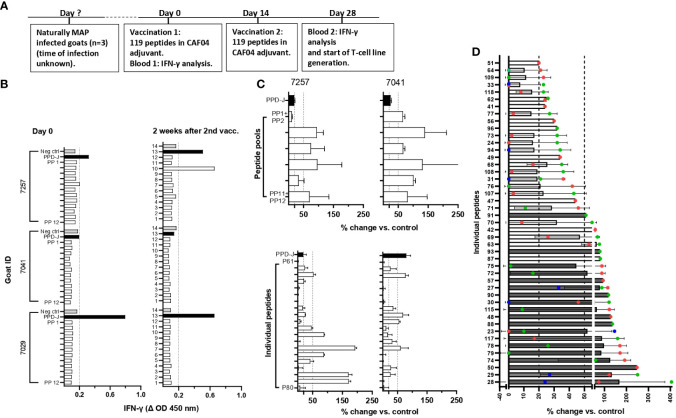
Screening for peptide recognition in naturally MAP-infected goats before and after peptide immunization. **(A)** Timeline showing when vaccination and blood analysis were performed. **(B)** IFN-γ responses after stimulation of heparin blood with peptide pools (PP1–PP12) at day 0 and 2 weeks after the second immunization of peptides formulated with CAF04. IFN-γ values at ΔOD ≥0.2 were regarded as a positive response (dotted line). **(C)** Upper: Percent change in proliferation of peptide pool-stimulated T-cell lines relative to unstimulated controls (% change vs. control) for two of the MAP-infected animals (mean ± standard deviation (SD) of tested duplicates). Lower: T-cell lines that showed reactivity toward the peptide pools were further expanded and tested for recall responses against individual peptides from the pools used to generate the T-cell line. The proliferation of T-cell lines from animals 7257 and 7041 after stimulation with individual peptides P61–P80 in peptide pools 7 and 8 is shown to illustrate an example. **(D)** Peptides inducing the strongest proliferation of the T-cell line is shown as mean ( ± SD) plus individual values in different colours (red, blue, and green) for three goats. For some peptides, it was not possible to generate T-cell lines from more than one animal. Dark gray bars represent peptides that were chosen for the immunization of healthy goats based on the strength of response and number of responding animals. Unpublished data for IFN-γ responses in peptide vaccinated cattle was also taken into account when peptides were selected for further testing. Proliferative T-cell line responses (% change vs. control) ≥20 to <50 were classified as medium responses, while values ≥50 were classified as strong responses, as indicated by the dotted lines in **(B, C)**. PP, peptide pools; PPD-J, purified protein derivative from MAP (Johnin).

MAP-infected goats were vaccinated with MAP-specific peptides and examined for antigen-specific IFN-γ and T-cell line responses (**Figure 2A**). No IFN-γ response was seen after stimulation of blood from three MAP-infected goats with pools of peptides prior to immunization (day 0) (**Figure 2B**). After two rounds of immunization using peptides formulated in CAF04, there was an IFN-γ response toward one of the 12 peptide pools in one animal; otherwise, no response to other peptide pools was detected (**Figure 2B**).

Due to the low responses when stimulating blood from vaccinated animals with peptide pools, it was decided to increase the sensitivity for detection of vaccine responses by producing T-cell lines. T-cell lines were first generated against PPDj to establish the protocol. The use of CD4-selected T-cell lines was incorporated as a standard in the protocol due to overgrowth of CD8+ and γδ T-cells in mixed T-cell lines (**Supplementary Figure S1A**). CD4-selected T-cell lines stimulated with PPDj had an average proliferation of 91.6% which was significantly higher than the response against *E. coli* sonicate -4.2% (**Supplementary Figure S1B**). Intracellular staining after PPDj stimulation of CD4-selected T-cell lines showed an increase in the percentage of IFN-γ producing CD4+ T-cells (**Supplementary Figure S1C**). These results indicated that the generation of T-cell lines against MAP antigens (PPDj) had been successful.

Next, CD4+-sorted T-cell lines were generated after cultivation with peptide pools. Most T-cell lines responded with proliferation when tested for recall responses to the peptide pools they were generated against (**Figure 2C**, upper). The T-cell lines also responded against several of the individual peptides (**Figure 2C**, lower). Based on the strength of proliferation and the number of animals with a T-cell line response against a specific peptide, the 19 peptides inducing the most promising T-cell response were chosen for a new immunization trial with healthy goats (**Figure 2D**; **Tables 1**, **2**). In addition, four other MHC-predicted or hydrophobic peptides (nos. 45, 103, 109, and 110), listed in **Table 1** or **Table 2**, were included for further studies as IFN-γ responses to these peptides had been detected following peptide vaccination of cattle (unpublished data from a trial not included in this paper). Thus, the final selection of peptides for further studies with healthy goats included 14 MHC-predicted peptides and nine hydrophobic peptides.

### IFN-γ, antibody, and T-cell line responses in healthy, peptide-vaccinated goats

**Figure 3:**
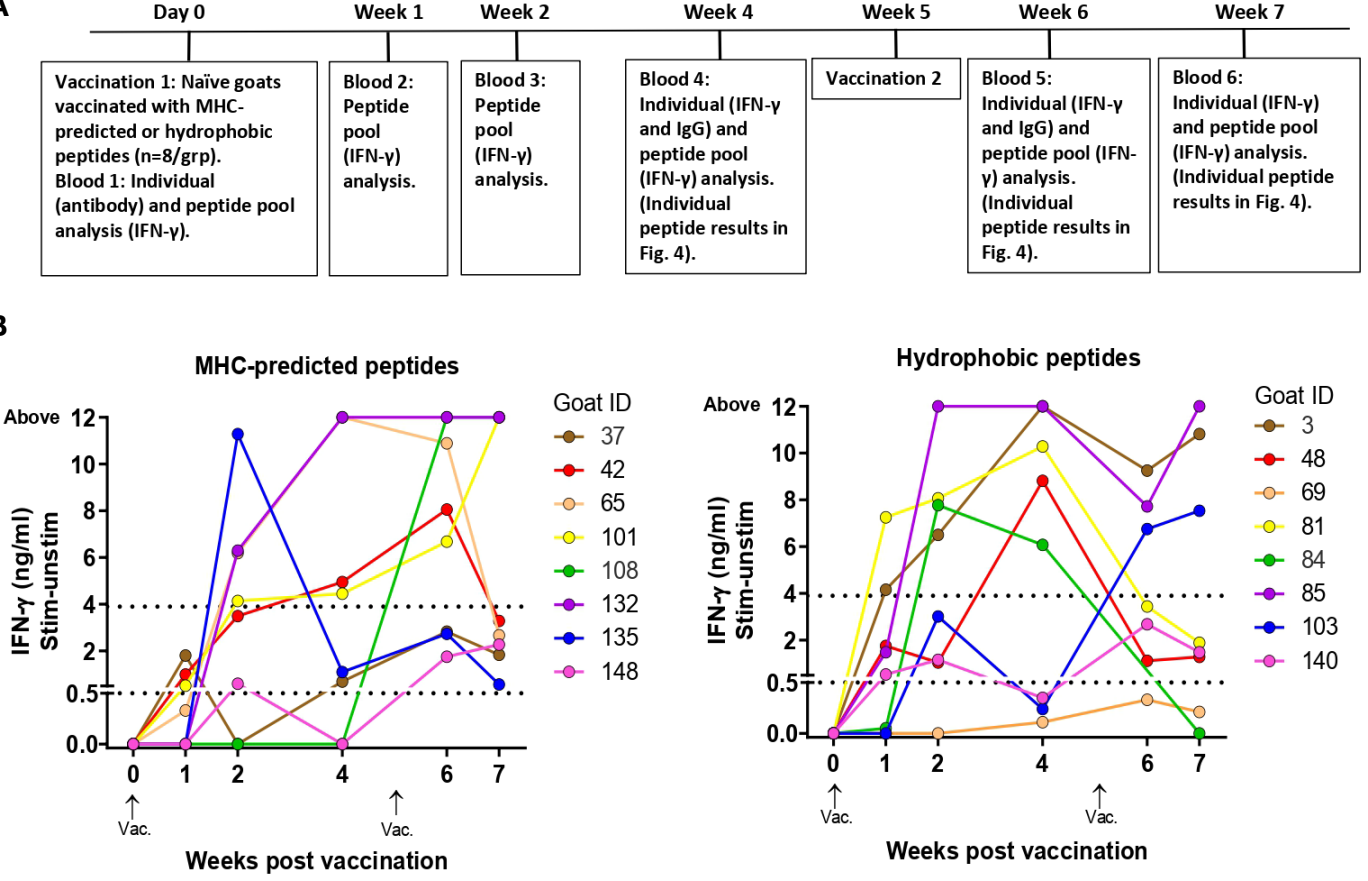
Immune responses to peptide pools in healthy, peptide-vaccinated goat kids. **(A)** Timeline showing when vaccination and blood analysis were performed. The timeline also indicates sampling for testing of individual peptide responses as shown in **Figure 4**. **(B)** IFN-γ responses to peptide pools were measured at day 0 and 1, 2, 4, 6, and 7 weeks after initial vaccination with MHC-predicted peptides (left) or hydrophobic peptides (right) formulated in Montanide ISA 61 VG. Blood was stimulated with a pool of all the peptides the goat had been immunized with. IFN-γ values ≥0.44 ng/mL were classified as positive responses, while values ≥3.9 were classified as strong responses (indicated with dotted lines).

Based on T-cell line responses of MAP-infected goats vaccinated with 119 peptides, healthy goat kids were vaccinated with 14 MHC-predicted peptides, nine hydrophobic peptides, or adjuvant only. Because of the low IFN-γ responses in the initial trial, CAF04 adjuvant was replaced with Montanide ISA61 VG in the remainder of this study. The goat kids were examined for antigen-specific IFN-γ, antibody, and T-cell line responses (**Figure 3A**).

IFN-γ responses to pools of injected peptides: IFN-γ responses toward the pool of all injected peptides were detected already at 1 week after vaccination, and after 2 weeks strong responses were seen in several of the goats (**Figure 3B**). From this time point and throughout the study, the responses were significantly different from day 0 for both groups (maximum *p*-value 0.0313). All goats in the MHC-predicted group and all except one goat in the hydrophobic group, had peptide pool responses above cutoff at two or more time points (**Figure 3B**). There was no significant difference between the two peptide groups at any time point after vaccination (minimum *P*-value = 0.1372). Three goats in the MHC-predicted group and four from the hydrophobic group were tested 3 months after revaccination. With the exception of one animal, all goats had IFN-γ responses toward the peptide pool above cutoff (mean ΔOD 1.28; range: 0.185–3.571). No IFN-γ response to peptide pools was seen at week 7 after initial vaccination in the group injected with adjuvant only, and the mean ΔOD was 0.002 (range: -0.028–0.041).

**Figure 4:**
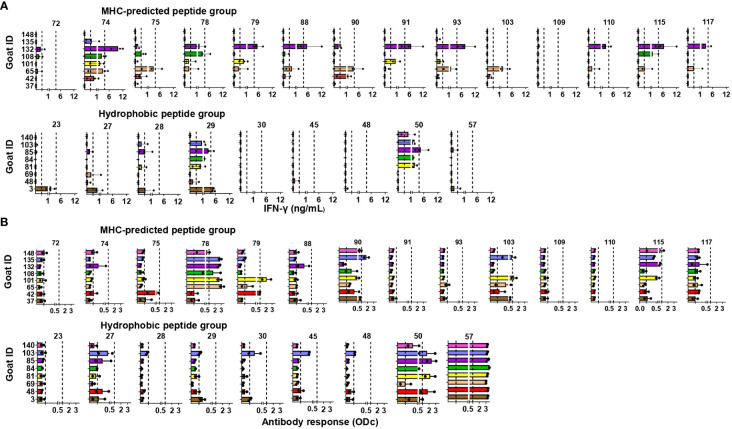
Immune responses to individual peptides in healthy, peptide-vaccinated goat kids. **(A)** IFN-γ responses to individual peptides were measured 4, 6, and 7 weeks after initial vaccination with MHC-predicted peptides (upper) or hydrophobic peptides (lower) formulated in Montanide ISA 61 VG by stimulation of blood with the individual peptides the animal had been vaccinated with. IFN-γ values ≥0.44 ng/mL were classified as positive responses, while values ≥3.9 were classified as strong responses (indicated with dotted lines). **(B)** Antibody responses measured by ELISA 4 and 6 weeks after vaccination with MHC-predicted peptides (upper) or hydrophobic peptides (lower) formulated in Montanide ISA 61 VG. Serum was analyzed using ELISA plates coated with the individual peptides the animal had been vaccinated with. The results in **(A, B)** are shown as mean and individual values for all time points analyzed after vaccination.

IFN-γ responses to individual peptides: At weeks 4, 6, and 7 after the vaccination of healthy goat kids, blood was stimulated separately with each of the individual peptides the animal had been immunized with. In the MHC-predicted group, one peptide did not induce IFN-γ production in any of the goats (P109). The other peptides gave a response above cutoff in a minimum of one and a maximum of six of the goats (**Figure 4A**). Three hydrophobic peptides (P30, P45, and P48) did not induce IFN-γ production in any of the goats. IFN-γ levels above cutoff were seen in between one and six of the goats for the remaining hydrophobic peptides, with P29 and P50 eliciting the strongest IFN-γ responses (**Figure 4A**). IFN-γ responses above cutoff were seen after peptide pool stimulation in some goats where no response to individual peptides could be detected (**Figures 3B**, **4A**).

Antibody responses to individual peptides: Strong IgG responses were seen against several of the MHC-predicted peptides when tested 4 and 6 weeks after initial vaccination. Two peptides (P91 and P110) did not induce an antibody response above cutoff, while antibodies were found against the other peptides in between one and eight goats (**Figure 4B**). One peptide (P28) did not induce a detectable IgG response above cutoff in any of the goats in the hydrophobic group. Two peptides (P50 and P57) stood out by inducing a moderate to strong IgG response in all the goats, while the rest of the hydrophobic peptides mounted an antibody response in one to five goats. Some responses were close to cutoff (**Figure 4B**). Antibody responses were seen in a higher number of animals compared to the number of goats with IFN-γ responses for eight of the MHC-predicted peptides and six of the hydrophobic peptides (**Figures 4A, B**). No antibody responses were seen prior to vaccination (mean ODc: 0.04; range: -0.6–0.22), except one goat having an ODc value of 0.22 (P109, MHC-predicted group) and another goat having a ODc value of 0.21 (P45, hydrophobic group). Additionally, no antibody response was seen in goats injected with adjuvant only (mean ODc: 0.074; range: -0.018–0.172).

**Figure 5:**
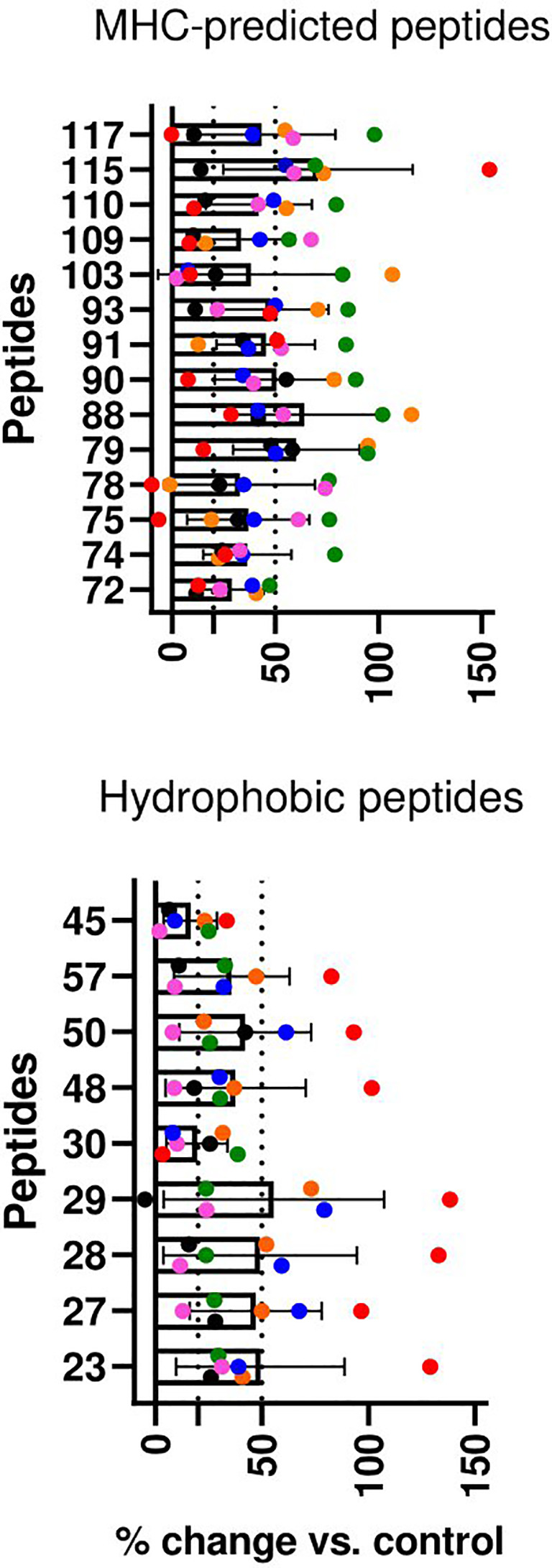
T-cell line responses to individual peptides in healthy, peptide pool-vaccinated goat kids. CD4+ T-cell lines were cultivated with and tested against all the peptides the animals had been vaccinated with. Percent change in proliferation for peptide-stimulated compared with unstimulated controls (% change vs. control) is shown as mean ( ± SD) and individual values for six T-cell lines from two goats in the MHC-predicted group (upper) and six T-cell lines from four goats in the hydrophobic group (lower). Individual values for each T-cell line are presented in different colors. T-cell line responses were classified as medium (≥20) or strong (≥50) as indicated by the dotted lines.

T-cell line responses to individual peptides: CD4-selected T-cell lines were made from two goats in the MHC-predicted group (six T-cell lines) and four goats from the hydrophobic group (six T-cell lines). A proliferative response above 50% for at least one T-cell line was seen with all the individual peptides, except one peptide in the MHC-predicted group (P72) and two peptides in the hydrophobic group (P30 and P45) that had proliferative responses between 20% and 50% (**Figure 5**).

### IFN-γ, antibody, and T-cell line responses in healthy, peptide-vaccinated cattle

**Figure 6:**
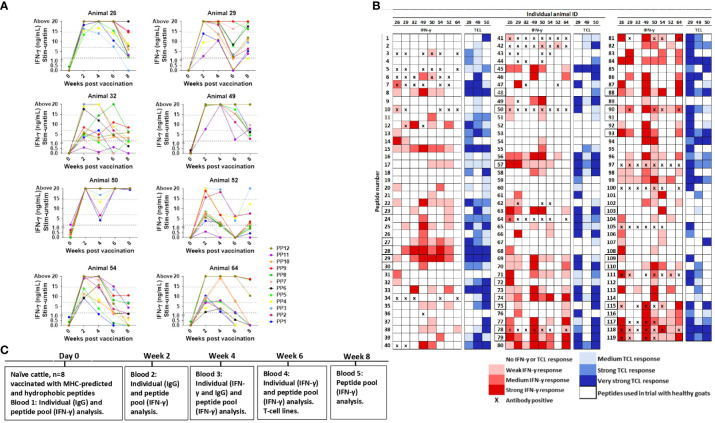
Immune responses in peptide-vaccinated heifers. **(A)** IFN-γ responses in heifers were measured at day 0 and 2, 4, 6, and 8 weeks after vaccination with all 119 peptides formulated in Montanide ISA 61 VG. Blood was stimulated separately with peptide pools (PP) consisting of nine to 10 peptides, where PP1-PP6 are hydrophobic peptides and PP7-PP12 MHC-predicted peptides. IFN-γ values ≥1.8 ng/mL were classified as positive responses. Values between 7 and 14.7 ng/mL were classified as medium responses, while values ≥14.7 were classified as strong responses (indicated with dotted lines). **(B)** Recognition of individual peptides in vaccinated heifers. Peptide numbers are shown in the left columns and animal ID in the upper row. The colors indicate the strength of IFN-γ response (shades of red, classified as in **(A)**), and the strongest IFN-γ response from testing at 4 and 6 weeks after vaccination is shown. T-cell line response is shown in shades of blue, and proliferation was classified as % change *versus* control as medium (≥20 to <50), strong (≥50 to <100), or very strong (≥100). Antibody ELISA results are indicated as positive (X) if the responses were detected in samples 2 and/or 4 weeks after vaccination. Peptides surrounded by a bold line were included in the 23 peptides used for the vaccination of healthy goat kids. **(C)** Timeline showing when vaccination and blood analysis were performed. TCL, T-cell line.

The testing of peptides in healthy goats had shown encouraging results. Since our initial *in silico* selection of peptides was based on binding to bovine MHC II molecules, we moved from screening and testing in goats to cattle. Healthy heifers were then vaccinated with a pool of all 119 peptides formulated with Montanide ISA61 VG and tested for immune responses to the peptides (**Figure 6C**).

IFN-γ responses to pools of injected peptides: Blood was stimulated separately with pools of nine to 10 peptides. With one exception, IFN-γ responses were below cutoff at day 0. Strong IFN-γ responses were generally detected from 2 weeks after vaccination (**Figure 6A**). The responses to all peptide pools were significantly different from day 0 at every time point after vaccination (*p* = 0.078–0.0313), except for PP1 at weeks 6 and 8 plus PP11 at week 6 (**Figure 6A**). No IFN-γ response to any of the peptide pools was seen in the animal injected with adjuvant only (mean: ΔOD 0.0045, range: -0.029–0.081).

IFN-γ responses to individual peptides: IFN-γ responses toward individual peptides were analyzed 4 and 6 weeks after vaccination. The majority of peptides (84%; 100/119) induced an IFN-γ response in at least one of the eight vaccinated heifers, and 18 MHC-predicted and nine hydrophobic peptides induced IFN-γ production in five animals or more (**Figure 6B**). In total, 23 of the peptides had a medium or strong response in at least three animals (**Figure 6B**).

Antibody responses to individual peptides: Peptide-specific IgG antibodies in at least one animal was found toward 29 of the individual peptides (24%) when tested 2 and 4 weeks after vaccination. Five of these peptides were not found to induce IFN-γ production in any animal, while 10 of them stimulated IFN-γ production in three or more of the animals (**Figure 6B**). Peptides classified as antibody inducers had ODc below cutoff at day 0 (mean ODc: 0.145; range: 0.053–0.145). The exception was responses to peptide number 97 in some animals, but the response after vaccination was at least seven times higher than before immunization.

T-cell line responses to individual peptides: T-cell lines were made from three vaccinated heifers. The proliferation of T-cell lines after stimulation with individual peptides was seen in one or more animal for 108 out of the 119 peptides, and for 40 of these peptides, proliferation was seen in all three animals. Proliferation of at least one T-cell line, but no IFN-γ response in any animal, was seen for 15 of the peptides. An IFN-γ response with no T-cell line proliferation was found for seven peptides, but four of these were peptides that induced IFN-γ response in just one animal (**Figure 6B**).

### Comparative immune responses to individual peptides in goats and cattle

**Figure 7:**
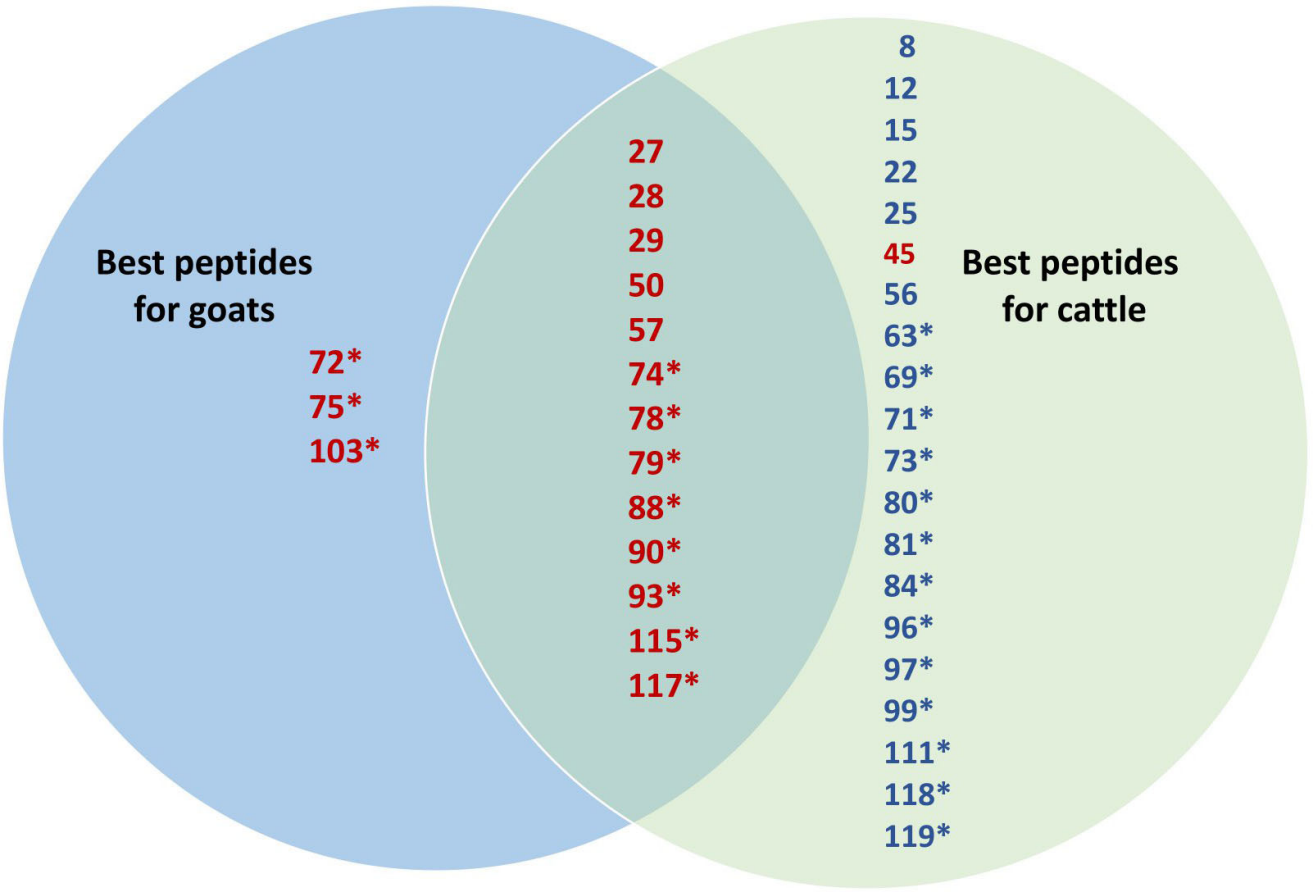
Venn diagram comparing the immune response to individual peptides in goats and cattle. The 23 peptides selected based on the proliferation of T-cell lines from MAP-infected, peptide-vaccinated goats were ranked from 1 (best) to 5 in both goats and cattle. The score was based on the number of animals with IFN-γ, TCL, and antibody responses in healthy goats and cattle. IFN-γ responses were weighted 2.5 times higher than antibody responses. As T-cell lines were not made from all animals, the TCL response was scored from 1 to 3 and added to the combined IFN-γ and antibody score. Only the best 17 peptides with a rank of 1–3 in both goats and cattle are shown in the figure in red and are allocated as useful for both or only one of the species in the Venn diagram. Based on a similar ranking, 19 additional peptides inducing a promising immune response after the vaccination of healthy cattle with all 119 peptides were identified. These additional 19 peptides with a high rank (1–3) in cattle are shown in blue. Peptides marked with an asterisk are MHC-predicted, while hydrophobic peptides are those which have no asterisk.

The 23 peptides selected for the vaccination of healthy goats as a result of T-cell line proliferation in MAP-infected, vaccinated goats were ranked from 1 (best) to 5. The score was based on the number of animals with IFN-γ and antibody responses in healthy goats and cattle. IFN-γ responses were weighted 2.5 times higher than antibody responses when scoring the peptides. As T-cell lines were not made from all animals, the TCL response was scored from 1 to 3 and added to the combined IFN-γ and antibody score. Out of the 23 peptides, 13 had a high rank (1–3) in both goats and cattle, three had a high rank in goats only, while one was mainly promising for cattle (**Figure 7**). Among the peptides with a high rank in both goats and cattle, 62% were MHC-predicted peptides and 38% were hydrophobic peptides. Based on a similar ranking as shown above, an additional 19 peptides inducing promising immune responses were identified after the vaccination of healthy cattle with all 119 peptides, out of which 68% were MHC-predicted peptides (**Figure 7**). These additional peptides were not tested in healthy goats with Montanide ISA61 VG adjuvant.

### Diagnostic testing for *M. bovis* and paratuberculosis

Skin testing of cattle was performed 8 weeks after vaccination to test if the peptide immunization could induce cross-reactivity with the SICCT test. None of the animals tested positive on the SICCT. The maximum increase in skin thickness at the PPDb site was 2 mm, and the difference in increase between the PPDb site and the PPDa never exceeded 1 mm. A single animal responded to the PPDa injection with a reaction of 5 mm (**Supplementary Table S3**).

To investigate if immunization with the identified MAP peptides interfered with antibody testing for paratuberculosis and *Mycobacterium bovis*, serum from cattle was analyzed with the ID Screen paratuberculosis Indirect ELISA and the IDEXX *M. bovis* antibody test. All peptide-vaccinated heifers tested negative on both tests (**Supplementary Tables S4**, **S5**).

## Discussion

This study aimed to identify and test immunogenic MAP-specific peptides which would not interfere with the diagnosis of bovine tuberculosis. Two panels of MAP-specific peptides were identified by comparing the MAP genome with other mycobacterial genomes. This was followed by *in silico* selection of MHC-II binding peptides with predicted high binding affinity (MHC-predicted peptides) or identification of hydrophobic peptides within proteins with previous knowledge of immunogenicity (hydrophobic peptides). The identified peptides were checked against peptides available in the IEDB, but no match was found, which supports the novelty of these peptides. Several immunogenic peptides were identified based on testing of CD4+ T-cell lines and measuring IFN-γ and antibody responses after the immunization of goats and cattle. Furthermore, no reactivity was seen when testing serum from vaccinated cattle with ELISA kits for bovine tuberculosis or paratuberculosis or when performing tuberculin skin testing. Taken together, the peptides appear promising for developing a peptide-based vaccine against paratuberculosis with reactivity toward a number of MAP proteins and without interfering with the diagnosis of *M. bovis*. However, challenge trials need to be performed in order to determine if the peptides can offer protection against MAP infection.

By using computer algorithms, the entire bacterial genome of an organism can be examined to predict peptide binding to MHC molecules. In contrast, experimental testing of all possible peptides in the MAP genome would be extremely laborious. Previous studies have demonstrated the appropriateness of NetMHCIIpan for effectively predicting binding to human leukocyte antigen (HLA)-DR molecules, even if exact information about the examined MHC molecule is lacking (52, 53). Furthermore, programs that predict HLA-DR binding have previously been used to identify peptides from *Mycobacterium bovis* that were recognized by bovine T-cells (54, 55). The bovine MHC complex is highly polymorphic with numerous DRB3 and DQB alleles listed in the IPD-MHC database. As the BoLA type of cattle used for immunization was unknown, the most promiscuous peptides among the predicted binders were selected to facilitate binding to various MHC class II genotypes. To justify the immunization of goats with peptides predicted to bind bovine MHC-II, the identified peptides were tested for predicted binding to goat MHC class II beta chains and found to bind promiscuously. Our results indicate that, by using NetMHCIIpan for predicting peptide binding to several bovine MHC-II molecules, one could successfully identify 15–18-mer MAP peptides that were immunogenic in both cattle and goats without matching the target animals to a specific MHC profile.

To increase the chance of identifying suitable vaccine candidates, hydrophobic peptides were also included during the initial peptide selection, in addition to peptides selected based on MHC prediction binding. Hydrophobicity was used as inclusion criteria as it has previously been shown that exposing hydrophobic domains can enhance antigen presentation by MHC molecules (56). Furthermore, hydrophobicity has been reported to correlate with MHC I immunogenicity as immunogenic epitopes have a predominance of hydrophobic amino acids at T-cell receptor contact residues (42). This is not fully investigated for MHC II-specific peptides, but the crystal structures of HLA-DR have demonstrated a hydrophobic binding cleft (57) and that the two primary cooperative peptide-binding pockets of HLA-DR are hydrophobic (58).

There was a predominance of MHC-predicted peptides compared to hydrophobic peptides among the 23 peptides selected for further testing of goats. When testing these 23 peptides in healthy goats and cattle with Montanide ISA 61 VG adjuvant, more MHC-predicted peptides were found to be strongly immunogenic compared to hydrophobic peptides. Additionally, the most promising candidates for the immunization of both cattle and goats were predominantly MHC-predicted peptides. This suggests that the MHC-predicted peptides had better species cross-reactivity than the hydrophobic peptides and that NetMHCIIpan might be used to estimate the level of MHC-binding cross-reactivity between cattle and goats. The immunization of cattle with the initial 119 peptides in Montanide ISA 61 V adjuvant identified more peptides as immunogenic compared to those found in goats. This might be due to the fact that another adjuvant (CAF04) was used in the initial goat studies or due to individual or species differences. Goats were not vaccinated with all 119 peptides in Montanide ISA 61 VG adjuvant, making it difficult to compare cattle and goat responses for other peptides than the 23 discussed. Another observation was that some peptides with consecutive numbers could all provoke an immune response in several cattle or goats, such as peptides 27–29, 77–81, and 117–119. In most cases, there is no obvious reason for this, and the observations could simply be due to chance. Peptides 27 and 28 and peptides 74–75, however, originate from the same gene, which could explain why these consecutively numbered peptides induced immune responses in several animals.

The cellular immune response to subdominant epitopes can be relatively low, and low or non-detectable responses were noted after the vaccination of MAP-infected goats with the 119 initially selected peptides. Hence, a protocol for the generation of CD4+ T-cell lines was established to evaluate the immunogenicity of these peptides. The method was adapted from a human protocol for establishing CD4 T-cell lines (10, 48) and included the isolation of CD4+ T-cells and enrichment of MAP antigen-reactive T-cells by cultivation, followed by testing for reactivity toward antigens. The protocol was established and optimized using PPDj as an antigen, and the success of the method was confirmed in that a majority of the generated CD4+ T-cell lines proliferated and produced IFN-γ in response to PPDj. Subsequently, the same protocol was used for the generation and screening of CD4+ T-cell lines from the blood of three MAP-infected goats immunized with MAP-specific peptides. It cannot be ruled out that these T-cell lines were partly elicited by the infection. However, the goal was to identify immunogenic peptides that would later be tested in immunization trials with healthy animals and subsequently for the ability to induce protection in challenge trials. Based on T-cell line responses, several promising peptides were identified as candidates to be included in further testing in goats. While some peptides induced the proliferation of T-cell lines in only one animal, other peptides were recognized by two or all three animals. These differences could be explained by individual MHC genotype, and the most promiscuous peptides would be particularly good candidates for inclusion in a future vaccine. Even though all the 119 peptides had been chosen based on *in silico* binding to MHC II or previous immunogenicity and hydrophobicity, not all peptides induced the proliferation of T-cell lines. This is a demonstration of the individuality of T-cell reactivity and highlights the advantage of combining *in silico* analysis with *ex vivo* generation of T-cell lines.

Strong IFN-γ responses were seen against several of the MAP-specific peptides after immunization, but the response pattern varied between animals. While the IFN-γ response increased after the second dose for some of the healthy goats, the response tended to decrease in others. Although it cannot be ruled out that the lack of a boosting effect was due to an unspecific activation of T-cells in response to peptide vaccination, the individual variation in IFN-γ responses suggests that this is unlikely. Fluctuating IFN-γ responses have been seen in MAP-infected animals (47, 59, 60) and could potentially also be found in vaccinated animals and complicate the interpretation of vaccine responses. A study testing heat-killed MAP in different adjuvants found reduced IFN-γ responses after the second dose when a formulation with Montanide ISA 61 V was used, while antigen-specific antibody responses increased. Vaccine formulations with other adjuvants resulted in increased IFN-γ responses upon boosting (61). As the choice of adjuvant seem to influence the immune response to vaccine antigens, we encourage future work to test different adjuvants.

In addition to the IFN-γ responses detected in the vaccinated, healthy goat kids, a predominant IgG antibody response was found toward several of the peptides in these animals. In cattle, however, fewer peptides induced antibody responses compared to IFN-γ responses. Cattle were provided one dose of vaccine, and goats were given two doses in our study. It has previously been shown that a second dose of heat-killed MAP in Montanide ISA 61 V reduced the IFN-γ response but had the potential to increase the antigen-specific antibody responses (61). Although antibody responses were detected after peptide vaccination in the current study, binding of the peptide-specific antibodies to the actual 3D conformational epitopes was not tested. A Th1 response with IFN-γ production has been considered essential for the development of protective immunity following MAP vaccination (1, 2); however, IFN-γ production is not always correlated with protection in MAP-vaccinated animals (2). Antibody responses have traditionally been considered to be of little significance for protection against MAP, but B-cells have been shown to decrease the mycobacterial tissue burden and reduce the immunopathology associated with *Mycobacterium tuberculosis* infection (62–64). Furthermore, a study using a heat shock protein 70 (Hsp70) vaccine for post-exposure vaccination against MAP indicated that antibody responses were more strongly associated with protection than Hsp70-specific IFN-γ responses (65). A study in sheep failed to correlate both IFN-γ responses and antibody levels to protection of MAP vaccinated sheep, while B-cell function appeared important (66). Further studies in sheep have shown that a strong, early IgG1 response after vaccination was central for protection against MAP infection (67). In addition, Th-17 responses that induce inflammation during early disease stages may also be important for protection against mycobacteria (32, 68–70). Most likely, a combination of humoral and cell-mediated immune responses would offer the best protection against MAP (29, 71), and future testing of the MAP peptides should ideally also include an analysis of other immune parameters (e.g., IL-17 and CD8+ T-cell responses).

It is likely that a high number of peptides must be included to produce a vaccine that is effective for both goats and cattle and to account for individual variations in immune responses to peptides and different expressions of MAP proteins during the course of infection. Future studies should ideally test different combinations of the candidate peptides in challenge trials with both species. The current study used synthetic peptide molecules in the immunization studies. However, the identified epitopes have the potential to be used in other delivery systems; for instance in mRNA vaccines which have been the leading vaccines against SARS-CoV-2 infection. Intranasal vaccination with naked mRNA-heat shock protein (Hsp65) from *Mycobacterium leprae* has shown protection against challenge with *Mycobacterium tuberculosis* (*M. tuberculosis*, MTB) in mice (72). Furthermore, vaccination against *M. tuberculosis* with replicating RNA formulated in a nanostructured lipid carrier has led to lower bacterial burdens in the lungs of mice after aerosol challenge with MTB compared to controls (73). Despite some evidence that RNA-based vaccines can offer protection against mycobacteria, vaccines against these complex bacteria likely require the inclusion of peptides or sequences from multiple proteins. However, it is not known how many different sequences can be included in an mRNA vaccine, and this should be addressed before deciding to use this vaccine platform.

The vaccination of MAP-infected goats with CAF04 liposome adjuvant did not result in injection site reactions, but IFN-γ responses were low. An additional attempt to vaccinate 30 healthy goat kids with peptides in CAF01 and CAF04 adjuvant also resulted in low IFN-γ responses (unpublished data), even though both CAF01 and CAF04 were expected to induce effective Th1 responses (45, 74). To improve responses in later trials with healthy cattle and goats, peptides were instead formulated with water-in-oil Montanide ISA 61 adjuvant. Vaccine formulations of heat-killed MAP with Montanide ISA 61 have previously resulted in less injection site reactions in sheep compared to vaccination with Gudair (61), but lesions were seen in up to 87.5% of vaccinated sheep when this adjuvant was combined with recombinant MAP antigens (75). In the present study, swelling and granuloma-like structures were seen in goats and cattle injected with the peptides in Montanide ISA 61. In the goat trial, no reactions were seen in goats injected with adjuvant only. The reactions thus appeared to be related to the combination of peptides with adjuvant. Large reactions were also seen in cattle following immunization. Although no infection was evident at the site of injection, the observations indicate that the vaccine was not optimal in terms of reducing adverse reactions compared to currently available MAP vaccines. Further studies should be conducted to reduce the injection site reactions, like testing lower doses, different injection sites, different adjuvants, or different vaccine delivery systems such as viral vector or possibly mRNA vaccines.

In conclusion, the approach of identifying immunogenic peptides by *in silico* analysis and generating T-cell lines to evaluate the immune response was confirmed as a valuable tool for selecting immunogenic peptides for use in a potential vaccine against paratuberculosis. Strong cellular and humoral immune responses were seen against several of the identified MAP-specific peptides after immunization, but with individual immunogenicity patterns between different animals. The results also indicate that the approach with selection of peptides unique to MAP successfully led to animals being vaccinated without interference with skin testing for bovine tuberculosis. These peptides thus show potential for inclusion in a new vaccine against MAP that would not interfere with surveillance and trade tests for bovine tuberculosis. Based on the proliferation of T-cell lines, IFN-γ, and antibody responses, it was possible to identify the most promising peptide candidates to be tested in different combinations in future challenge trials with goats, cattle, or other ruminants. The peptide sequences identified and described in this paper could also be tested in other vaccine modalities, such as viral vectors or potentially mRNA, to both obtain increased immune response as well as reduced observed adverse effects at the site of injection.

## Data availability statement

The original contributions presented in the study are included in the article/**Supplementary Material**. Further inquiries can be directed to the corresponding author.

## Ethics statement

All animal experiments were approved by the Norwegian Animal Research Authority according to the EU directive 2010/63/on the protection of animals used for scientific purposes: FOTS ID 3871 (MAP-infected goats trial), FOTS ID 5375 (healthy goats trial), and FOTS ID 8509 (healthy cattle trial). The study was conducted in accordance with the local legislation and institutional requirements.

## Author contributions

KL: Data curation, Formal analysis, Investigation, Methodology, Software, Writing – original draft, Validation, Conceptualization, Visualization. ST: Conceptualization, Data curation, Formal analysis, Investigation, Methodology, Project administration, Validation, Writing – review & editing, Supervision, Visualization. HM: Investigation, Methodology, Validation, Writing – review & editing. CL: Software, Data curation, Methodology, Validation, Writing – review & editing, Conceptualization, Funding acquisition, Resources. SS: Formal Analysis, Visualization, Writing – original draft, Methodology, Validation, Writing – review & editing, Data curation, Investigation. CA: Conceptualization, Funding acquisition, Methodology, Validation, Writing – review & editing, Data curation, Resources, Visualization. IO: Conceptualization, Data curation, Formal analysis, Funding acquisition, Investigation, Methodology, Project administration, Validation, Writing – review & editing, Supervision, Visualization. GJ: Conceptualization, Data curation, Formal analysis, Funding acquisition, Investigation, Methodology, Project administration, Validation, Writing – review & editing, Supervision, Visualization.
