# Supplementary material for: Selection of vaccine-candidate peptides from Mycobacterium avium subsp. paratuberculosis by in silico prediction, in vitro T-cell line proliferation, and in vivo immunogenicity
Source: Front Immunol. 2024 Jan 30;15:1297955. doi: 10.3389/fimmu.2024.1297955 (PMC10861761; doi:10.3389/fimmu.2024.1297955)
Supplement: Supplementary file 1 [file Presentation_1.pdf]

Supplementary material

Supplementary table 1. Study design, sampling and analyses for vaccine trials.

	Vaccine trials		
	**Naturally MAP infected goats**	**Healthy goat kids**	**Healthy heifers**
**Groups and animals**	3 adult goats.<br>Infection was confirmed by positive culture, PCR and IFN-γ result.	24 healthy, castrated, male goat kids.<br>Three groups of 8 animals.	9 heifers
**Peptides for vaccination**	59 MHC-predicted peptides and 60 hydrophobic peptides injected in all animals	Groups:<br>1) 14 MHC-predicted peptides<br>2) 9 hydrophobic peptides<br>3) Adjuvant only	59 MHC-predicted peptides and 60 hydrophobic peptides injected in 8 heifers.<br>1 heifer injected with adjuvant only
**Vaccinations**	Day 0 and week 4	Day 0 and week 5	One injection at day 0
**Dose and injections sites**	50 µg /peptide/goat, s.c., neck (2mL)<br>MHC-predicted and hydrophobic peptides administered at separate sides of the neck	20 µg/peptide/goat, s.c, axialla (2 mL)	20 µg/peptide/heifer, s.c, neck (2 mL)<br>MHC-predicted and hydrophobic peptides administered at separate sides of the neck
**Adjuvant**	CAF04: 100 µl of peptides mixed with 0.5 mL adjuvant and subsequently added 1.4 mL of 10 mM Tris (hydroxymethyl) aminomethane buffer (pH 8.0).	Montanide ISA61 VG (volume ratio of 4:6 of peptide: adjuvant).	Montanide ISA61 VG (volume ratio of 4:6 of peptide: adjuvant).
**IFN-γ assay**	• Day 0 and 2 weeks after 2nd immunization.<br>• Stimulation with 12 peptide pools (PP) of 9-10 peptides in separate wells.	• Day 0 and 1,2,4,6, and 7 weeks post initial vaccination.<br>• Stimulation with all the peptides the goat had been vaccinated with as a single peptide pool.<br>– Adjuvant group stimulated with both MHC-predicted and hydrophobic peptides.<br>• Stimulation with all peptides the animal had been vaccinated with individually in separate wells (only 4, 6 and 7 weeks post vaccination).	• Day 0 and 2, 4, 6 and 8 weeks post vaccination<br>• Stimulation with 12 peptide pools (PP) of 9-10 peptides each in separate wells.<br>• Stimulation with all 119 peptides individually in separate wells (only 4 and 6 weeks post vaccination).
**ELISA for detection of antibodies to peptides**	Not done	• Day 0, week 4, and week 6 post initial vaccination<br>• In house ELISA coated with individual peptides	• Day 0, week 2, and week 4 post vaccination<br>• In house ELISA coated with individual peptides
**MAP ELISA (IDscreen)**	Not done	Not done	0, 2, 4 and 6 weeks after vaccination
**M. bovis ELISA (IDEXX)**	Not done	Not done	0, 2, 4 and 6 weeks after vaccination
**Tuberculin skin test**	Not done	Not done	8 weeks after vaccination
**T-cell lines (TCL)**	• Mixed and CD4 -selected TCL cultivated with PPDj, and testing against PPDj and *E.coli*.<br>• CD4-selected peptide reactive TCL generated from two weeks after 2nd vaccination.<br>– Cultivation with peptide pools.<br>– Testing against peptide pools and individual peptides.	• T-cell lines made from two goats in the MHC-predicted group and four from the selected group.<br>• CD4-selected peptide reactive TCL generated from two months after 2nd vaccination.<br>– Cultivation with the peptide pool the goat had been vaccinated with.<br>– Testing against peptide pools and individual peptides.	• CD4-selected peptide reactive TCL generated from three animals 6 weeks after vaccination.<br>– Cultivation with peptide pools.<br>– Testing against peptide pools and individual peptides.

S.c.=subcutaneous, w=week, TCL=T-cell line

Supplementary table 2. Overview of antibodies used for flow cytometry and generation of T-cell lines.

Antibody	Clone	Final concentration (µg/mL)
CD4[1]	GC1A (goat)	10*
CD4[1]	ILA11A (cattle)	7.7*
CD8[1]	CACT80C	1
γδ T-cell receptor [1]	GB21A	5
IFN-γ[2]	CC302	5
APC-conjugated goat anti-mouse[3]		5
FITC-conjugated goat anti-mouse[3]		10
PE-conjugated goat anti-mouse[3]		5

*1µg/10x10^6 cells for sorting.

Allophycocyanin (APC), Fluorescein isothiocyanate (FITC), Phycoerythrin (PE).

Producers are listed in regard to superscript; 1. Monoclonal antibody center, USA. 2. Bio-Rad, USA. 3. Southern Biotech, USA.

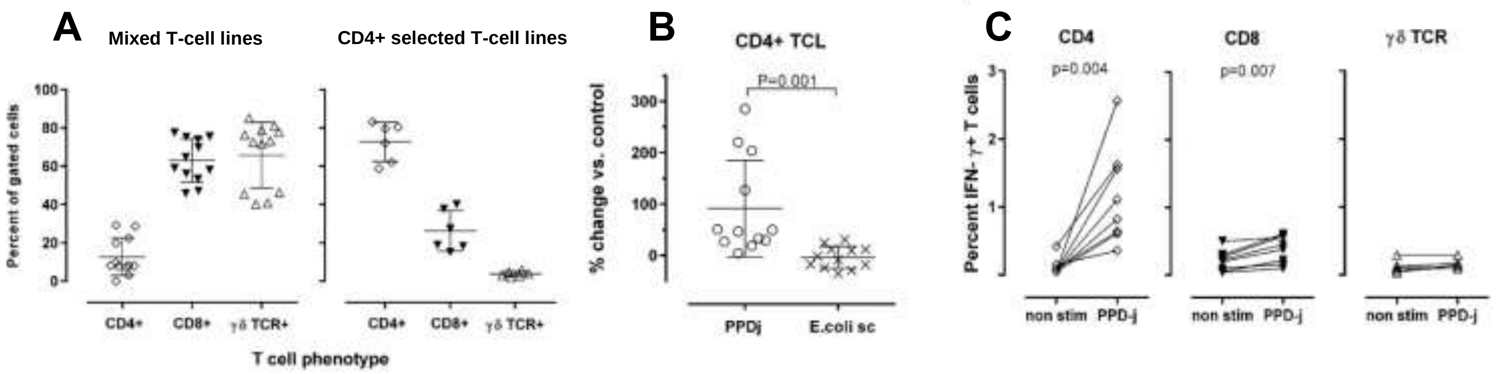

Supplementary figure 1.

Establishment of protocol for generation of T-cell lines using PPDj. T-cell lines were made from three MAP-infected goats. **(A)** Flow cytometric phenotyping of T-cell lines for CD4, CD8 or γδ TCR after two weeks of cultivation. The figure shows results in percent positive cells (mean +/- SD) for mixed T-cell lines (left) and CD4+ selected T-cell lines (right). Each symbol represents one T-cell line. **(B)** Percent change in proliferation (mean +/- SD) of PPDj and *E. coli* sonicate stimulated cells compared with unstimulated controls for CD4+ selected T-cell lines. Each symbol represents one cell line. **(C)** Intracellular staining for IFN-γ production showing percent IFN-γ positive CD4, CD8 and γδ TCR cells for unstimulated and PPDj stimulated T-cell lines. Two symbols connected with a line represent cells from one T-cell line.

Supplementary table. 3 Results from the single intradermal comparative cervical tuberculin (SICCT) test of cattle performed eight weeks after vaccination with MAP-specific peptides.

		After 72 hours				
Animal ID	Skin thickness time of injection (mm)	Skin thickness PPD_B (mm)	Skin thickness PPD_A (mm)	Increase PPD_B (mm)	Increase PPD_A (mm)	PPD_B minus PPD_A
26	10	10	10	0	0	0
29	9	10	10	1	1	0
32	10	10	10	0	0	0
49	10	10	11	0	1	-1
50	7	7	7	0	0	0
52	8	9	11	1	3	-2
54	7	9	12	2	5	-3
64	9	10	9	1	0	1

Supplementary table. 4 Results from the ID Screen paratuberculosis indirect ELISA at day 0 and 2, 4, and 6 weeks after vaccination of cattle with MAP-specific peptides. Results are shown as SP% with interpretation as follows: Positive: SP% >70. Doubtful: 60 < SP% < 70. Negative: SP% < 60.

	Individual animal ID							
	**26**	**29**	**32**	**49**	**50**	**52**	**54**	**64**
**Day 0**	6,2%	6,0%	3,5%	4,7%	7,8%	11,0%	10,1%	24,8%
**Week 2 post vaccination**	7,8%	11,8%	8,1%	7,6%	11,1%	14,0%	11,9%	28,0%
**Week 4 post vaccination**	7,1%	11,1%	6,3%	6,5%	10,9%	12,6%	9,5%	27,4%
**Week 6 post vaccination**	5,8%	12,0%	8,7%	6,6%	10,7%	10,8%	10,4%	27,2%

Supplementary table. 5 Results from IDEXX *M. bovis* antibody test at day 0 and 2, 4, and 6 weeks after vaccination of cattle with MAP-specific peptides. Results are shown as S/P ratio. Positive results are S/P ratio >0.3.

	Individual animal ID							
	**26**	**29**	**32**	**49**	**50**	**52**	**54**	**64**
**Day 0**	-0.076	-0.057	-0.084	-0.057	-0.061	-0.064	-0.058	-0.076
**Week 2 post vaccination**	-0.078	-0.064	-0.079	-0.074	-0.032	-0.075	-0.067	-0.066
**Week 4 post vaccination**	-0.064	-0.060	-0.055	-0.044	-0.064	-0.082	-0.046	-0.072
**Week 6 post vaccination**	-0.059	-0.037	-0.069	-0.062	-0.051	-0.074	-0.061	-0.043
